# Efficacy evaluation of multi-immunotherapy in ovarian cancer: From bench to bed

**DOI:** 10.3389/fimmu.2022.1034903

**Published:** 2022-10-06

**Authors:** Xiaoyi Hu, Ce Bian, Xia Zhao, Tao Yi

**Affiliations:** Department of Gynecology and Obstetrics, Development and Related Disease of Women and Children Key Laboratory of Sichuan Province, Key Laboratory of Birth Defects and Related Diseases of Women and Children, Ministry of Education, West China Second Hospital, Sichuan University, Chengdu, China

**Keywords:** ovarian cancer, immunotherapy, multi-immunotherapy, immune checkpoint inhibitor, adoptive cell therapy, cancer vaccine, oncolytic virus

## Abstract

Ovarian cancer, one of the most common gynecological malignancies, is characterized by high mortality and poor prognosis. Cytoreductive surgery and chemotherapy remain the mainstay of ovarian cancer treatment, and most women experience recurrence after standard care therapies. There is compelling evidence that ovarian cancer is an immunogenic tumor. For example, the accumulation of tumor-infiltrating lymphocytes is associated with increased survival, while increases in immunosuppressive regulatory T cells are correlated with poor clinical outcomes. Therefore, immunotherapies targeting components of the tumor microenvironment have been gradually integrated into the existing treatment options, including immune checkpoint blockade, adoptive cell therapy, and cancer vaccines. Immunotherapies have changed guidelines for maintenance treatment and established a new paradigm in ovarian cancer treatment. Despite single immunotherapies targeting DNA repair mechanisms, immune checkpoints, and angiogenesis bringing inspiring efficacy, only a subset of patients can benefit much from it. Thus, the multi-immunotherapy investigation remains an active area for ovarian cancer treatment. The current review provides an overview of various clinically oriented forms of multi-immunotherapy and explores potentially effective combinational therapies for ovarian cancer.

## 1 Introduction

Ovarian cancer is the most lethal gynecological malignancy, of which epithelial ovarian cancer (EOC) is the most prevalent subtype. Most EOC patients are diagnosed with advanced stage accompanied with tumor spread to the peritoneal cavity. Current frontline treatments include debulking surgery, platinum-taxane maintenance chemotherapy, and recently developed targeted agents and immunotherapy. Despite aggressive treatment, the 5-year survival rate for women diagnosed with stage III or IV disease is still less than 25% (1). Most patients would suffer a recurrence after the initial response to therapy and almost all of them resistance to chemotherapy and leading to the death.

**Figure 1:**
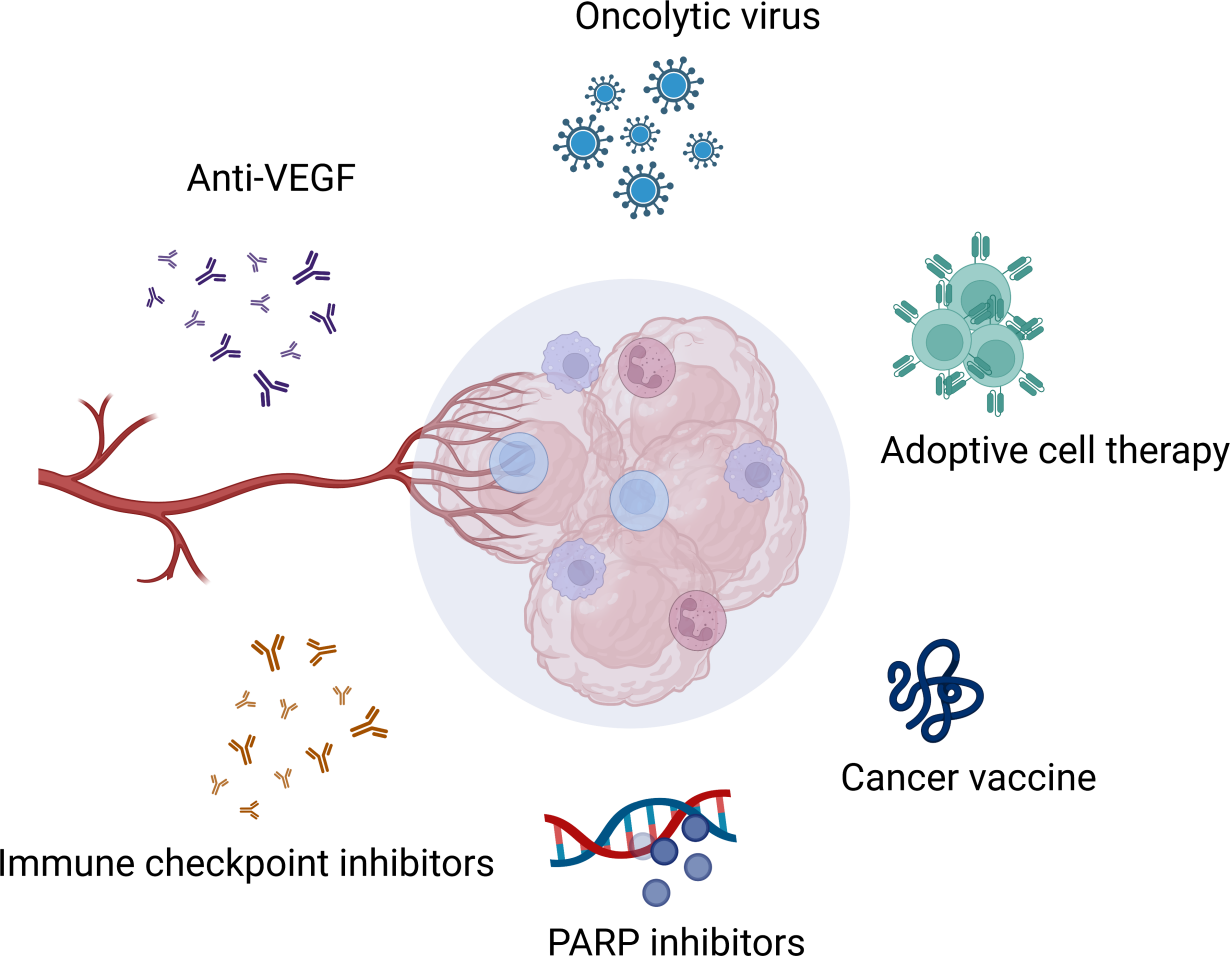
Immunotherapies in ovarian cancer. Created with BioRender.com.

Growing evidence suggests that ovarian cancer is immunogenic cancer. There has been a significant increase in understanding of molecular and genetic changes in the ovarian cancer microenvironment. Thus, various immunotherapies target the tumor microenvironment (TME) and attempt to address the challenges posed by the highly immunosuppressive TME (2). Current immunotherapy for ovarian cancer includes immune checkpoint blockade, adoptive cell therapy, cancer vaccine, oncolytic virus and so on (**Figure 1**). Despite several of them achieving inspiring efficacy in the clinic, such as PARP inhibitors. Only a tiny fraction of patients benefited from them, and most of them would eventually suffer a recurrence or progression. With the limited efficacy brought by studies testing single-agent immunotherapy in recurrent ovarian cancer, optimism has resurfaced around the possibility that combinational therapy would deliver the better outcome expected by the community. In this review, we summarize the progress of clinical developments in multi-immunotherapies for ovarian cancer and briefly discuss the future directions of combinational therapies in ovarian cancer.

## 2 Tumor microenvironment in ovarian cancer

**Figure 2:**
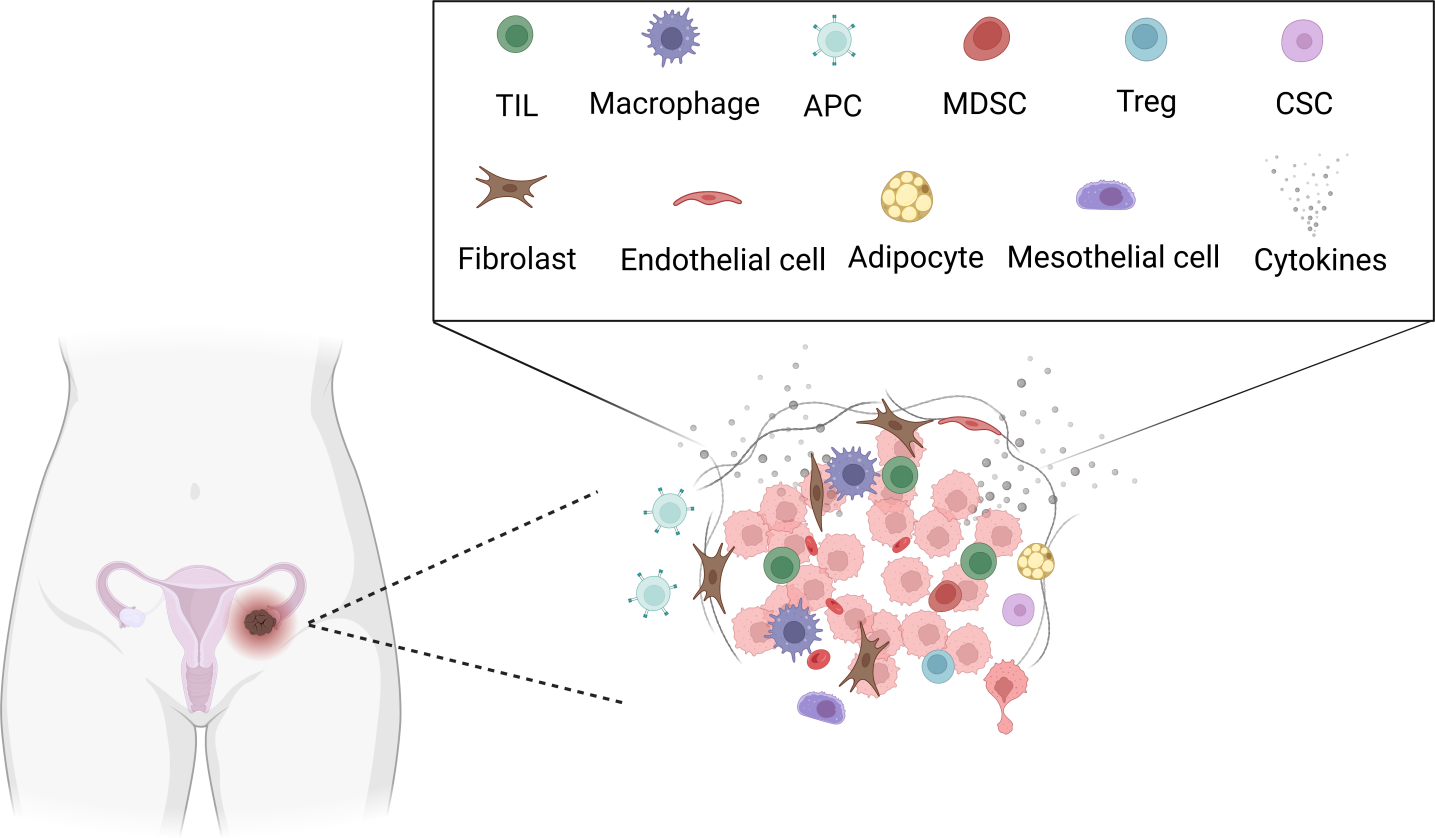
Tumor microenvironment in ovarian cancer. Created with BioRender.com. TIL: Tumor-infiltrating lymphocytes (TILs), APC: Antigen-presenting cell, MDSC: Myeloid-derived suppressor cells (MDSCs), Treg: Regulatory T, CSC: cancer stem cell.

The TME comprises the extracellular matrix (ECM) and stromal cells. The ECM consists of water, proteoglycans, minerals, and fibrous proteins secreted by resident cells in an interlocking network (3). The ECM plays a critical role during tumorigenesis, affecting cell migration, invasion, and metastasis. Besides, stromal rearrangement plays a supportive role during the malignancy progresses and eventually, the tumoral and stromal changes aggravate each other and promote a dynamic reciprocity cycle (4). The matrix-centric, stromal-targeted cancer therapies developed as the ECM is altered at the biochemical, architectural, biomechanical, and topographical levels (5). Stromal cells in the TME include cancer-associated adipocytes, mesothelial cells, fibroblasts, and immune cells. Immune cells include tumor-infiltrating lymphocytes (TILs), Tregs, neutrophils, macrophages, dendritic cells (DCs), natural killer (NK) cells, myeloid-derived suppressor cells (MDSCs), polymorphonuclear neutrophils (PMNs), and so on (6, 7) (**Figure 2**). The tumor-permissive TME is achieved by reprogramming host cells to support tumor phenotypes and functions (6). The metastatic tropism of cancer cells to the omentum, characterized by highly vascularized immune cell structures called milky spots, plays a critical role in the generation of the metastatic TME in the intraperitoneal cavity (6). In addition, not only components in the TME communicate and impact each other, but also ovarian cancer cells communicate with TME through various signaling pathways, such as STATs family pathway, IL-6 pathway, and NF-KB pathway (1). Several factors are associated with response to immunotherapy, including T cell exhaustion, PD-L1 status, microsatellite instability, mismatch repair deficiency, Tumor mutation burden (TMB), CD8+ positivity, T cell infiltration and so on (8). Thus, immunotherapies target TME developed, current immunotherapies target ovarian cancer TME including CAFs targeting therapy, anti-angiogenesis therapy, immune checkpoint inhibitors (ICIs), oncolytic virus and so on (9). Tumors responsive to ICIs are usually called hot tumors, which depends on T cells’ infiltration. On the contrary, cold tumors usually do not respond to ICIs, which is characterized by poor T cell infiltration (10). Besides, the effectiveness of immunotherapy is associated with baseline immune responses and unleashing of pre-existing immunity. Thus, combinational immunotherapies may boost weak antitumor immunity, enhance tumor antigens cross-presentation, and promotes T cell priming and infiltration (11).

## 3 Targeting DNA repair-based combination immunotherapies

There are at least five recognized pathways that exist for DNA repair: direct repair, mismatch repair (MMR), nucleotide excision repair (NER), base excision repair (BER), and double-strand break (DSB) recombinational repair. DSB occurs by non-homologous end-joining and high-fidelity homologous recombination repair, which is much more error prone (12). Besides, germline aberrations in critical DNA repair and DNA-damage response (DDR) genes contribute to cancer susceptibility syndromes, including BRCA1, BRCA2, BLM, FANCA, TP53, RAD51C, and MSH2. After exposure to carcinogens, the generation of DNA damage increases the risk of cancer. Therefore, genomic instability is a recognized hallmark of cancer (13). Various agents are developed to target different processes during DNA repair, including PARP inhibitors, NER inhibitors, BER inhibitors, DDR kinases inhibitors, inhibitors targeting termini recognition, end bridging, DNA-end processing, and DNA ligation, inhibitors targeting homology directed repair and Rad51 (14). We will focus on PARPi-based combinational therapies, as it is most widely studied in ovarian cancer.

### 3.1 PARPi-based combination immunotherapies

The poly (ADP-ribose) polymerase (PARP) is a recognized sensor of DNA damage, which is known for its role in DNA BER and DNA single-strand breaks (SSB) repair. The role of PARP in DSB repair is less elucidated (13). PARP inhibitors have been a new targeted treatment for ovarian cancer, particularly in women with BRCA1 and BRCA2 mutation or patients without a functional homologous recombination repair pathway (15). Homologous recombination deficient cells are susceptible to PARP inhibitors. BRCA1 and BRCA2 are tumor suppressor genes. They are associated with fundamental roles in DNA repair by forming a homologous recombination repair complex (16). Several PARP inhibitors are approved by the US Food and Drug Administration (FDA) or studied in clinical trials, including olaparib, niraparib, rucaparib, veliparib, and talazoparib (17). On March 27, 2017, niraparib was approved by the US FDA. The approval is based on the results of NOVA (NCT01847274) (18). On April 6, 2018, the US FDA approved rucaparib for the maintenance treatment. The approval relies on ARIEL3 (NCT01968213) (19, 20). Based on the results of SOLO-1 (NCT01844986), on December 19, 2018, the US FDA approved olaparib for the maintenance treatment of adult patients with germline or somatic BRCA-mutated (gBRCAm or sBRCAm) who exhibited either a complete or partial response to first-line platinum-based chemotherapy (21). Nevertheless, a recent clinical trial indicated that the efficacy of platinum-based subsequent chemotherapy seems to be reduced in BRCA1/2-mutated patients with platinum-sensitive relapsed ovarian cancer (PSROC) compared to patients who haven’t received PARPi therapy (22). Despite the inspiring benefits PARPi brought, lots of limits still exist. Future studies should focus more on combinations that can enhance the effect of PARPi, benefit patients with non-HRD tumors, mitigate toxicity, and overcome PARPi resistance (23). Therefore, the combination of PARPi and other immunotherapies are developed, especially antiangiogenic agents and immune checkpoint inhibition.

#### 3.1.1 PARPi combined with antiangiogenic agents

Angiogenesis plays a vital role in normal ovarian physiology as well as in ovarian cancer pathogenesis. Tumor progression and growth largely depend on angiogenesis, as tumor could not grow beyond 1-2 mm if the neovascularization cannot meet the requirements of nutrients and oxygen. Thus, antiangiogenic agents have been incorporated into the therapy regimen for ovarian cancer. Vascular endothelial growth factor (VEGF) and VEGF receptor (VEGFR) are primarily explored in clinical settings, and this pathway contributes to malignant ascites and tumor progression (24). Besides, it is also shown that overexpressed VEGF is correlated with tumor staging and prognosis (25). Plenty of angiogenesis inhibitors are being investigated, including Bevacizumab, Aflibercept, Nintedanib, Cediranib, Pazopanib, Sunitinib, Sorafenib, and Trebananib (26). Approved by the FDA, Bevacizumab exhibited modest efficacy, and most patients developed acquired resistance. Therefore, the combination of PARPi and angiogenesis inhibitors are reasonable and meaningful.

There are two purposes for combining PARPi and angiogenesis inhibitors. Firstly, PARPi could decrease angiogenesis (27). Secondly, both VEGF3 inhibitors and hypoxia induce the downregulation of HRD proteins (28, 29). On May 8, 2020, the indication of olaparib was expanded to combination therapy with bevacizumab for first-line maintenance treatment of HRD-positive advanced ovarian cancer (30). The approval was based on the PAOLA-1 trial, which revealed that combined therapy of bevacizumab and olaparib provided a significant progression-free survival (PFS) benefit in HRD-positive patients, regardless of whether the patient had the BRCA mutation (31). More combinational strategies are being studied. In a patient-derived ovarian cancer xenografts (OC-PDXs) model, the combination of PARPi Olaparib and VEGFR inhibitor cediranib reduced the growth of all OC-PDXs independent of BRCA status (32). In 2014, a phase 2 study revealed that Cediranib plus Olaparib could prolong PFS (33). Later, a phase 3 clinical study NRG-GY004 showed that combining Cediranib and Olaparib did not prolong PFS compared with chemotherapy and resulted in reduced patient-reported outcomes (PRO) (34). Besides, other combinational strategies are being investigated too. Compared to monotherapy, niraparib plus bevacizumab significantly increased the PFS of platinum-sensitive recurrent ovarian cancer, while a more extensive scale phase 3 clinical trial is planned (35, 36). More preclinical and clinical studies are needed to provide information about the most appropriate combination strategy and which subset of patients in what clinical setting benefit most.

#### 3.1.2 PARPi combined with immune checkpoint inhibitors

In addition to antiangiogenic agents, PARPi was combined with other targeted immunotherapies, such as PD-1/PD-L1 inhibitors, WEE-1 inhibitors, ataxia-telangiectasia-mutated-and-Rad3-related kinase (ATR) inhibitors, MEK inhibitors, and so on (37). Plenty of studies regarding PARPi and PD-1/PD-L1 combinational therapy are completed or ongoing. Olaparib, niraparib, rucaparib, and talazoparib are combined with anti-PD-1 antibodies (nivolumab, pembrolizumab) and anti-PD-L1 antibodies (durvalumab, atezolizumab, avelumab) (38). PARPi and PD-1/PD-L1 antibodies demonstrated synergistic antitumor activities in animal models regardless of BRCA mutation status, which is achieved by blockade of single-stranded DNA damage repair and activation of the STING-dependent immune response. Moreover, PARPi induces an immunostimulatory micromilieu in ovarian cancer, thereby complementing the activity of PD-1/PD-L1 blockade (39, 40). A phase 2 clinical trial revealed that a combination of olaparib and durvalumab showed modest efficacy whereas blockade of VEGF/VEGFR would be necessary to improve the combination (41). PARPi was also combined with many other ICB in ovarian cancer, such as inhibitors target phosphatidylinositol-4,5- bisphosphate 3-kinase (PI3K) (42, 43), V-akt murine thymoma viral oncogene homolog (AKT) (44), ATR (45, 46), heat shock protein 90 (HSP90) (47, 48), checkpoint kinase 1 (CHK1) (49), cytotoxic T-lymphocyte-associated protein 4 (CTLA-4) (50), salt-inducible kinase 2 (SIK2) (51), insulin-like growth factor-1 receptor (IGF-1R) (52). However, most of the combinations are still in preclinical or phase 1 clinical studies, and a larger scale of clinical studies is needed to further evaluate the efficacy. In addition, the natural compound alantolactone (ALT) could inhibit the thioredoxin reductase, thus inducing ROS accumulation and oxidative DNA damage in cancer cells. A combination of pro-oxidative agent ALT and Olaparib induced tumor regression, which broadened the application of PARP inhibitors (53).

Other agents targeting DNA repair are much less investigated in ovarian cancer. Some studies report their application in other types of cancers as previously reviewed (14). More data are needed on ovarian cancer.

## 4 Adoptive cell therapy-based combination immunotherapies

Adoptive cell therapy (ACT) mainly refers to chimeric antigen receptor (CAR)-modified T cells, T-cell receptor (TCR)-engineered T cells, natural TILs, CAR-NK cells, and CAR-macrophages. ACT has achieved a remarkable revolution in the hematological tumor. Nevertheless, for solid tumors, including ovarian cancer, ACT seems insufficient to elicit significant antitumor activity. In ovarian cancer, CAR-T cells target folate-receptor alpha (FRα), mesothelin, MUC-1, and HER2 have been widely investigated. However, no satisfactory therapeutic efficacy has been observed so far. The low avidity and heterogeneous expression of targetable membrane antigens and difficulties in CAT-T cell infiltration and survival are the key obstacles (54). Novel targets or combinational therapies are expected to solve these problems. For instance, CAR-T cells targeting the Mullerian inhibiting substance type 2 receptor (MISIIR), B7-H3, Epithelial cell adhesion molecule (EpCAM), C-X-C chemokine receptor 1 (CXCR1), or C-X-C chemokine receptor 2 (CXCR2), 5T4 significantly controlled tumor growth *in vivo (* 55–59). Apart from CAR-T therapy, other ACT, including TCR-T and CAR-NK, are also under investigation. TCR-T therapy is MHC restricted and relies on the presentation of the MHC complex. Unlike CAR-T therapy, whose target antigens are only cell surface proteins, TCR-T could recognize both intracellular antigen fragments and surface proteins as long as MHC molecules present them. In ovarian cancer, TCR-T targeting melanoma-associated antigen 4 (MAGE-A4) and New York esophageal-1 (NY-ESO-1) are in early clinical trials (60). CAR-NK targeting folate receptor alpha (αFR) (61), glypican-3 (GPC3) (62), human leukocyte antigen G (HLA-G) (63), CD44 (64), CD24 (65), CD133 (66), MSLN (67) have achieved therapeutic efficacy in preclinical studies. More clinical data are needed to verify their efficacy in ovarian cancer patients.

### 4.1 Bispecific CAR-T cells

As we mentioned, a common mechanism of tumor escape from single-target CAR-T cells is the downregulation and mutational loss of the targeted antigen. Thus, targeting multiple antigens may improve the efficacy of CAR-T cells. Several bispecific CAR-T products are under investigation. For instance, Zhen et found that folate receptor 1 (FOLR1) and mesothelin (MSLN) are specifically highly expressed in ovarian cancer cells by screening the GEO database. Therefore, they established tandem CAR-T cells target both FOLR1 and MSLN, and the tandem CAR-T cells exhibited enhanced antitumor activity and prolonged mouse survival compared to single-target CAR-T cells (68). Besides, MSLN CAR-T-secreting anti-CD40 antibody had a more powerful cytotoxic effect on ovarian tumor (69). Dual targeting tumor-associated glycoprotein 72 (TAG-72) and CD47 are effective in ovarian cancer model (70). CAR-T cells targeting PDL1 and MUC16 also demonstrated more potent antitumor efficacy than single-target CAR-T cells (71). Dual CAR-T cells targeting NKG2D and PD-1 ligands exhibited inspiring efficacy in treating metastatic peritoneal tumors (72). In the clinic, CAR-T cells targeting MSLN and PD-1 combined with apatinib exhibited potent therapeutic efficacy in one patient with refractory EOC (73). To summarize, most bispecific CAR-T therapies in ovarian cancer are still in the preclinical stages. Future studies should search for more specific and practical targets in the clinic.

### 4.2 CAR-T combined with other immunotherapies

According to the modest efficacy of CAR-T in ovarian cancer, several agents are applied to enhance CAR-T cells’ efficacy. Firstly, the efficacy of ICIs limited by a lack of a tumor-reactive microenvironment. CAR-T cells may provide the necessary tumor-targeting immune infiltrate. Conversely, ICIs counteract the immunosuppressive environment that undermines optimal CAR-T cell efficacy (74). Thus, combining ICI with CAR-T could be a promising strategy. By loading anti-HER2 or anti-EGFR bispecific antibodies, CD19-CAR-T and activated T cells showed comparable specific cytotoxicity against ovarian cancer cells (75). In addition, arm CAR-T cells with therapeutic cytokines. For instance, IL-12 secreting 4H11-28z CAR-T cells showed enhanced proliferation and antitumor ability compared to 4H11-28z CAR-T cells only (76). Besides, pretreatment of ovarian cancer cells with histone deacetylase inhibitor sodium valproate (VPA) could upregulate NKG2DL expression in ovarian cancer cells expressing low to moderate NKG2DL. Consequently, chimeric NKG2D CAR-T cells exhibited better efficacy by enhanced immune recognition (77). In some papers, upregulation or downregulation of certain receptors could enhance CAR-T cells’ efficacy. Co-expressing of CXCR2 enhanced homing and efficacy of CAR-T cells targeting the integrin αvβ6 (78). Besides, adenosine 2A receptors (A2aRs) disruption improved the efficacy of CAR-T cells targeting MSLN (79). As we mentioned before, poor T cell infiltration contributes to the failure of CAR-T therapy. Therefore, to improve T cell infiltration in ovarian cancer, a vascular disrupting agent (VDA) called combretastatin A-4 phosphate (CA4P) was combined with CAR-T cells and results indicated that CA4P enhanced the efficacy of CAR-T cells and could be an effective antitumor agent candidate in treating solid tumor (80). In addition, a substantial body of work suggests that the accumulation of adenosine in the TME contributed to the failure of immunotherapies. As a result, adenosine deaminase 1 (ADA) overexpression improved CAR-T cells’ antitumor ability in ovarian cancer (81). In summary, CAR-T-associated combinational therapy is still preclinical studies, and more reasonable and effective combinational strategies are being exploited.

### 4.3 Other ACT combinational therapies

CAR-NK, TCR-T and CAR-macrophage therapy are alternate cell-based therapies. Cancer-testis antigens (CTA) are developed as targets for TCR-T, including MAGE-A4 and NY-ES0-1 (60). CAR-NK offers some significant advantages compared to CAR-T, such as better safety, multiple cytotoxic mechanisms, and high feasibility for “off-the-shelf” manufacturing (82). CAR-NK against human leukocyte antigen G (HLA-G) inhibited tumor growth *in vitro* and *in vivo*, and such efficacy was enhanced when combined with chemotherapeutic agents (63). Besides, CXCR1 expression could enhance the antitumor efficacy of NKG2D CAR-NK, which provided a novel strategy for improving the therapeutic efficacy of NK cells (83). CAR-Macrophage own unique advantages. CAR-macrophage could significantly immerse in the TME, and direct kill tumor cells as well as enhance T cell function. In addition, CAR-macrophage has fewer non-tumor toxicities compared to CAR-T (84). Most CAR-macrophage therapies are in the preclinical stage, including CAR-macrophage targeting CD19, CD22, HER2, CCR7 and so on. Only several phase 1 clinical trials for solid tumors are ongoing (85). In ovarian cancer, reports of CAR-NK, TCR-T, and CAR-macrophage are rare. More data from preclinical and clinical studies are needed to prove the safety and antitumor efficacy.

## 5 Cancer vaccine-based combination immunotherapies

A single application of cancer vaccine in ovarian cancer is under exploration, such as peptide vaccine, whole tumor cell vaccine, cancer stem cells (CSCs), antigen-presenting cell (APC) vaccine, DNA/RNA vaccine, bacteria vaccine and so on. Most of them augment antitumor immunity in ovarian cancer patients. Nevertheless, clinical data only revealed modest efficacy in most patients. Therapeutic efficacy in more patients is testable (86–92). Despite most cancer vaccines only achieving moderate efficacy in other malignancies, combining cancer vaccines and other immunotherapies may broaden its application and elevate efficacy. For instance, murine ovarian cancer cell ID8 was spray dried and made into a microparticulate vaccine. The microparticulate ovarian cancer vaccine exhibited the most efficacious in inhibiting tumor growth when administered with interleukins (93). Adding immunomodulator agents such as IL-12 may augment the efficacy of cell-based cancer vaccine (94). In a phase 2 trial, a multiepitope FRα vaccine called TPIV200 was combined with PD-L1 inhibitor durvalumab in treating advanced platinum-resistant ovarian cancer. The combination was safe and elicited robust FRα-specific immune responses (95). Dual blockade of PD-1 and CTLA-4 enhanced efficacy of the GVAX vaccine in ovarian cancer models through activation of CD4 and CD8 T cells, secretion of cytokines, and inhibition of Treg cells (96). Besides, immunostimulatory adjuvant could elevate the efficacy of cancer vaccines. For instance, cowpea mosaic virus (CPMV) co-delivered with irradiated ovarian cancer cells elicited prophylactic efficacy and immunologic memory responses in mice models (97). 21 recurrent high-grade serous ovarian cancer (HGSOC) patients were treated with a polyvalent antigen-KLH plus OPT-821 vaccine and bevacizumab. Results indicated that the combinational therapy was well-tolerated. Although immunogenic responses were not associated with improved survival, researchers discovered that increased IL-18 correlated with improved PFS while increased PDGF was associated with worse OS (98). Gemogenovatucel-T (Vigil) is an autologous whole tumor cell vaccine transfected with GM-CSF gene and silenced of furin, the critical convertase responsible for activation of TGFβ-1 and TGFβ-2. The vigil was well-tolerated, but the primary endpoint was not met (99). A combination of vigil and a PD-L1 blocking antibody atezolizumab was safe. Further clinical exploration was justified (100). Apart from peptide and irradiated tumor cell vaccine, DC vaccine was combined with ex vivo-stimulated autologous T cells. Six patients were enrolled in this study. They received bevacizumab plus autologous DC pulsed with tumor lysate supernatants, followed by lymphodepletion and adoptive transfer of autologous vaccine-primed and CD3/CD28-stimulated T cells. Four patients benefit from the therapy, including two partial responses (PR) and two stable disease (SD) (101). Combining human monocytes and IFN-α2a and IFN-γ mediated potent antitumor effect in ovarian cancer (102). Immuno-modulators, including anti-CD40Ab and TLR3 ligand—poly(I:C), could enhance the antitumor effect of a DNA vaccine encoding MSLN and antigen-specific connective tissue growth factor (CTGF) (103). CPMV *in situ* vaccination combined with CD47-blocking antibody promoted macrophage activity and enhanced T cell function in ovarian cancer model (104). To summarize, most cancer vaccines could not wholly eradicate established tumors. They exhibit better therapeutic effects when tumor volume is small and the vaccine is given in an adjuvant setting (105).

## 6 ICI-based combination immunotherapies

### 6.1 Bispecific ICIs

Dual inhibition of PD-1/PD-L1 exhibited better efficacy in ovarian cancer compared to single-target. Bispecific targeting of PD-1 and PD-L1 induced superior cellular changes in T and NK cells compared to monospecific targeting (106). Besides, A soluble form of the PD-1 receptor (sPD-1) neutralized both PD-L1 and PD-L2 and achieved better efficacy. PD-L2 blockade facilitates ICB resistance through incomplete blockade of the PD-1 signaling pathway (107).

More inhibitors simultaneously target two signaling pathways to enhance the antitumor effects. APCS-540, a newly developed inhibitor targeting glycogen synthase kinase 3 beta (GSK3B) and histone deacetylases (HDACs), inhibited tumor growth and prolonged survival in an ovarian cancer model (108). Another inhibitor, Istiratumab, bispecific targets IGF-1R and epidermal growth factor receptor 3 (ErbB3). Istiratumab could be a candidate for treating chemotherapy-resistant ovarian cancer (109). Besides, MSC2363318A is a newly developed inhibitor targeting AKT1, AKT3, and P70S6K. Yes-associated protein (YAP1) could be a marker that predicts ovarian tumors’ sensitivity to MSC2363318A (110). HKMTI-1-005 simultaneously inhibited the histone methyltransferase G9A and EZH2, which elicited antitumor efficacy in HGSOC (111). Several papers focus on the pro-tumorigenic microenvironment induced by chemotherapy. Tumor cell debris produced by platinum- and taxane-based chemotherapy stimulates a “surge” of macrophage-derived proinflammatory cytokines and bioactive lipids. A dual cyclooxygenase-2 (COX-2) and soluble epoxide hydrolase (sEH) inhibitor PTUPB decreased proinflammatory cytokines and lipids in the TME and delayed ovarian tumor growth (112).

### 6.2 Dual blockade

When certain ICI works, it is possible that a compensatory signaling pathway was induced, providing an idea of the dual blockade. As one of the most widely applicated inhibitors, PD-1/PD-L1 inhibitors are combined with various inhibitors. Dual blockade of CXCL12-CXCR4 and PD1-PDL1 enhanced antitumor effects compared with the single blockade, which was associated with increased effector T cells infiltration and function, increased memory T cells, and decreased Treg cells in the TME (113). Dual blockade of PD-1 and CTLA-4 elicited antitumor efficacy in preclinical studies (114). A combination of PD-1 inhibitor Nivolumab and CTLA-4 inhibitor Ipilimumab in EOC patients resulted in superior responses and longer PFS (115). PD-1 inhibitor LY3300054 and CHK1 inhibitor prexasertib combinational therapy were tolerable and demonstrated preliminary efficacy in HGSOC patients (116). PD-L1 inhibitor atezolizumab and VEGF inhibitor bevacizumab achieved durable responses and/or disease stabilization in some platinum-resistant ovarian cancer patients (117). High expression of CXCL13 predicted a more prolonged survival and facilitated the maintenance of CXCR5+CD8+ T cells. Besides, CXCL13, combined with anti-PD-1 therapy, significantly retarded ovarian tumor growth (118). Combining cyclin-dependent kinases 4 and 6 (CDK4/6) inhibitor abemaciclib and anti-PD-1 therapy may have a better promise for poorly immune-infiltrated ovarian cancer (119).

Despite that more than 60% of ovarian cancers are positive for the estrogen receptor (ER), ER-targeted treatment in ovarian cancer was disappointing. Src is also activated in most ovarian cancers. It was found that estrogen could activate Src to phosphorylate p27, thus promoting its degradation and increasing cell-cycle progression. Combinational ER and Src blockade therapy by fulvestrant and saracatinib increased cell-cycle arrest, induced autophagy, and inhibited ovarian cancer growth *in vivo* (120, 121). Apart from Src inhibitor, MEK inhibitor selumetinib could also reverse antiestrogen resistance in ER-positive HGSOC. Besides, MAPK overexpression predicted poor prognosis and may help identify MEK inhibitor-responsive cancer (122).

Although the EGFR signaling pathway is usually activated and associated with a poor prognosis, clinical results of EGFR inhibition in recurrent ovarian cancer patients are disappointing. An article revealed that STAT3 activation might contribute to resistance to EGFR inhibition. Therefore, combined inhibition of EGFR and JAK/STAT3 had synergistic antitumor effects, whereas combinational inhibition of other pathways, including AKT/mTOR, MEK, and SRC, was relatively less effective (123). 12 patients received intraperitoneal cisplatin, intraperitoneal TLR3 ligand rintatolimad, and oral COX-2 blocker celecoxib. The study revealed that the combination was safe and tolerable. A phase 2 clinical trial would be tested (124). The insulin growth factor 1 (IGF-1) expression was elevated in two ovarian cancer models treated with bevacizumab. Dual blockade of IGF-1 and VEGF resulted in increased tumor growth inhibition (125). Delta-like ligand 4 (Dll4), one of the Notch ligands, is overexpressed in ovarian cancer. Dual blockade of Dll4 and VEGF markedly reduced ovarian cancer cell growth (126). Overexpression of BCL2L1 was associated with platinum resistance to multiple anti-cancer agents in ovarian cancer. Dual inhibition of FGFR4 and BCL-xL demonstrated potent efficacy and tolerable toxicity (127). Forkhead domain inhibitor-6 (FDI-6) is a forkhead box protein M1 (FOXM1). FDI-6 inhibition elicited the upregulation of N-Ras, phosphoprotein kinase Cδ (p-PKCδ), and HER3. Combination FDI-6 with tipifarnib (N-Ras inhibitor), rottlerin (p-PKCδ inhibitor), or sapitinib (HER3 inhibitor) decreased the survival of cancer cells (128). Src and MAPK are activated in HGSOC. Dual blockade of Src and MAPK by saracatinib and selumetinib inhibited ovarian tumor growth and targeted tumor initiating stem-like cells (129). Dual inhibition of DNA methylation and histone H3 lysine 9 dimethylation by 5-aza-CdR and G9Ai increased viral mimicry and served as a basis for this combination strategy (130). Combined inhibition of MEK and BCL-2/X_L_ had therapeutic efficacy in HGSOC models, and BIM protein was a biomarker of responsiveness (131). Dual inhibition of PI3K/mTOR and RAS/ERK by PF-04691502 and PD-0325901 showed robust synergistic antitumor efficacy (132).

Targeting agents participating in cancer cell metabolism are being explored. Dual inhibition of glycolysis and glutaminolysis could be a promising therapeutic strategy in ovarian cancer (133). Similarly, A triphenylphosphonium-modified terpyridine platinum (II) complex (TTP) inhibited multiple mitochondrial and glycolytic bioenergetics, thus inducing a hypometabolic state in several cancers, including ovarian cancer (134).

Besides EOC, research on other types of ovarian cancer was much less. The PI3K and murine double minute 2 (MDM2) upregulation predict a worse outcome in clear cell ovarian carcinoma (CCOC). Dual inhibition of PI3K and MDM2 by DS-7423 and RG7112 significantly reduced CCOC growth (135).

### 6.3 ICIs combined with other immunotherapies

Although ICIs have changed the practice of cancer treatment and prognosis, the application of ICIs for ovarian cancer is limited. Adding cytotoxic cytokines or neutralizing immunosuppressive cytokines may augment the efficacy. IL-10 in the TME sustained the immunosuppression in ovarian cancer. Therefore, IL-10 neutralization enhanced the antitumor efficacy of PD-1 blockade, and the combinational therapy prolonged survival and decreased tumor burden through T cell and B cell immunity in mice (136). Besides, active immunotherapy precedes administrated of ICI. Thus, promoting T cell maturation and resistance to the cytotoxic effects of the Bcl-2 inhibitor (137).

## 7 Oncolytic virus-based combination immunotherapies

Oncolytic viruses are gene-modified or naturally occurring viruses that selectively replicate and destroy cancer cells without harming the normal tissues (138). Adenovirus, herpes simplex virus (HSV), poxvirus, and measles virus are the most well-known oncolytic viruses in cancer therapy (105, 139). The oncolytic virus is combined chiefly with ICB in ovarian cancer. For example, oncolytic Maraba virus and PD-1 blockade combination mediated heterogeneous radiologic patterns through non-invasive MRI scanning (140). Plant virus CPMV nanoparticles conjugated with anti-PD-1 peptide had superior efficacy against metastatic ovarian cancer compared to adding free anti-PD-1 peptide (141). Oncolytic vaccinia virus therapy in ovarian cancer induced expression of PD-L1 in cancer cells and immune cells. Therefore, combining therapy of oncolytic vaccinia virus and PD-L1 blockade could synergistically enhance therapeutic efficacy (142).

Moreover, oncolytic viruses could be genetically modified to express exogenous cytokines or proteins. A modified Vaccinia Ankara vaccine expressing wild-type human p53 (p53MVA) promoted T cell responses, and combination with gemcitabine or other agents was expected to exhibit superior clinical responses (143). In addition, the oncolytic vaccinia virus (VV) engineered to express a fusion protein of IL-15 and IL-15Ralpha was named vvDD-IL15-Rα. A combination of vvDD-IL15-Rα and PD-1 blockade exhibited a dramatic tumor regression (144). Mice were pretreated with three homologous thrombospondin type 1 repeat domains (3TSR) alone or followed by combination with a fusogenic oncolytic Newcastle disease virus (NDV). 3TSR could normalize tumor vasculature, thus enhancing NDV delivery and trafficking of immune cells to the tumor core. The combinational therapy resulted in a most significant reduction in tumor volume and ascites accumulation (145).

Oncolytic viruses are also combined with other immunogenic agents. The oncolytic vaccinia virus (OVV) was enhanced by MEK inhibitor PD0325901 and trametinib in doxorubicin-resistant ovarian cancer (146). Microtubule destabilizing agents (MDAs) could sensitize tumors to oncolytic virus therapy. The combination of trastuzumab emtansine and oncolytic vesicular stomatitis virus (VSVΔ51) demonstrated that a viral-sensitizing molecule could enhance oncolytic virus efficacy (147). Infection of RNA virus induced upregulation of heat shock protein 70 (HSP70). HSP70 increased measles virus cytotoxicity. HSP90 inhibitors could upregulate HSP70, therefore increasing the efficacy of measles virotherapy (148). Furthermore, modulating interferon modulators by JAK1/2 inhibitor ruxolitinib could overcome partial resistance of an oncolytic vesicular stomatitis virus variant pseudotyped with the nonneurotropic glycoprotein (VSV-GP) (149).

The combination of two types of viruses demonstrated enhanced efficacy. For example, infection with Semliki Forest virus-ovalbumin (SFV-OVA) followed by infection with vaccinia virus-ovalbumin (VV-OVA) induced an enhanced antitumor efficacy through a combination of viral oncolysis and antigen-specific immunity (150).

A limitation of recombinant oncolytic virus therapy is the viral clearance by neutralizing antibodies. Therefore, a study found that cyclooxygenase-2 (Cox-2) inhibitors may circumvent this limitation. Cox-2 inhibitors successfully inhibited the generation of neutralizing antibodies and exhibited more effective antitumor efficacy when combined with the vaccinia virus in ovarian cancer (151). Another obstacle to viral therapy is that oncolytic viruses are large particles. Thus, it is difficult to efficient extravasation from tumor blood vessels. A study proved that the oncolytic sindbis virus target tumor cells by the laminin receptor. Therefore, modulating vascular leakiness by VEGF or metronomic chemotherapy could enhance specific targeting and delivery of sindbis viral vectors (152). Combination of adeno-associated virus (AAV) expressing 3TSR and Fc3TSR and bevacizumab extended mice survival, suggesting a further investigation of such a combination (153). The application of adenoviruses is limited by rapid, systemic cytokine release and consequently inflammatory toxicity. To overcome this obstacle, researchers used β3 integrin to significantly reduce toxicity without compromising antitumor efficacy (154).

## 8 Chemotherapy-based combination immunotherapies

Chemotherapy combined with cytoreductive surgery is the mainstay treatment for ovarian cancer. Although the majority of people initially respond to platinum-based chemotherapy, most patients would suffer a recurrence within 5 years. Currently, most clinical studies regarding immunotherapies are applied to patients who previously received chemotherapy, as we discussed before (37). Resistance to platinum agents and PARP inhibitors is one of the main obstacles to ovarian cancer therapy (155). Thus, it’s urgent to explore novel targets or combinational strategies. RNA sequencing and panel DNA sequencing revealed that neoadjuvant chemotherapy induces genomic and transcriptomic changes, and combined treatment of AP-1 or SIK2 inhibitors with carboplatin or paclitaxel showed synergistic effects (156). RNA sequencing analysis also suggested that stress promoted chemoresistance, which provided targets to overcome chemo resistance (157). In addition, targeting LRRC15 could inhibit metastatic dissemination through β1-integrin/FAK signaling (158). Apart from preclinical studies, several clinical trials revealed that MEK inhibitor trametinib, Wee1 inhibitor adavosertib, and CDK4/6 inhibitor ribociclib showed preliminary efficacy in ovarian cancer (159–161). Overall, a single application of immunotherapy is unlikely to have a dramatically effect in ovarian cancer. Understanding the interplay between signal pathways may provide a better combined therapy of chemotherapy and immunotherapy.

## 9 Immunotherapy enhancement strategy

### 9.1 Nanoparticles-based combination immunotherapies

Poor aqueous solubilities limited the application of several drugs. Nanoplatforms could help solve the barrier. Diblock copolymer nanoplatforms were used to formulate micelles through the solvent evaporation method. A dual drug loaded micelles (DDM) containing chetomin and everolimus targeted HIF and mTOR. The DDM significantly inhibited angiogenesis and induced apoptosis compared to the individual micells (162). Besides, ovarian tumor cells overexpress low-density lipoprotein receptors (LDLr). Thus, LDL-encapsulated cholesterol-conjugated heat shock protein 27 (HSP27) and human epidermal growth factor receptor 2 (HER2) dual inhibitor specifically targeted and inhibited ovarian cancer cells (163).

### 9.2 Radiotherapy-based combination therapy

Radiotherapy was nearly abandoned in ovarian cancer due to its modest efficacy and toxicity. However, recent studies revealed that a low dose of radiotherapy might reprogram the tumor microenvironment and reverse tumor immune desertification and resistance to immunotherapy (164). Low-dose radiotherapy plays a role in immune modulation and tumor microenvironment reprogramming rather than direct tumor killing. Although radiotherapy could promote antitumor immunity, including tumor antigen presentation and T cell recruitment, immune suppressive cells, including Tregs and MDSCs, are also activated. Therefore, radiotherapy combined with immunotherapy may promote the activity of favorable immune cells and elevate antitumor efficacies (164). Low dose radiotherapy (LDRT) triggered T cell infiltration in an IFN-dependent manner in ovarian cancer patients with immune-desert tumors when combined with immune checkpoint blockade (165). In a preclinical setting, radiation therapy combined with immunostimulatory CPMV elicited significant tumor retardation and increased TIL in the TME (166). Radiotherapy combined with immunotherapy in other types of cancers, including melanoma, lung cancer, and colon cancer, is under plenty of preclinical and clinical studies, providing a basis for application in ovarian cancer (164).

## 10 Conclusion and future perspectives

Ovarian cancer, especially epithelial ovarian cancer, is typically diagnosed at an advanced stage. Patients who experience a recurrence within six months after the end of platinum-based chemotherapy are characterized by poor prognosis, which needs a novel and effective treatment modality (167). Multi-immunotherapies are expected to prolong the survival and improve the prognosis, plenty of clinical trials are investigating their efficacy in ovarian cancer (**Table 1**). Immunotherapy could be strengthened through several points. Firstly, it is recommended that all women with newly diagnosed ovarian cancer should be offered genetic testing. Approximately 10%-20% of ovarian cancers are related to germline mutations. Besides, relatives of women with genetic mutations are recommended to have gene testing (168). In addition, several preclinical and early clinical data suggested that toll-like receptor 7 (TLR7) and TLR8 agonists could activate DCs, monocytes, macrophages, and fibroblasts. TLR7/8 agonists also promoted proinflammatory cytokines and chemokines secretion, including IL-6. Thus, activation of TLR7/8 may be a potential target (169). Moreover, RNA-associated therapy aroused researchers’ attention. Long non-coding RNAs (lncRNAs) are critical regulators in ovarian cancer occurrence and progression (170). RNA-binding proteins (RBPs), a class of endogenous proteins that bind to mRNA, regulate a series of pathological processes in ovarian cancer (171). Therefore, both lncRNAs and RBPs could be a potential therapeutic target (172–178). Non-coding RNA miR-146b simultaneously inhibited EGFR and IL6-STAT3 signal pathways, resulting in a more excellent suppression of ovarian cancer cell migration (179). Another non-coding RNA, HOTAIR, was overexpressed in ovarian cancer stem cells (OCSCs). Inhibition of HOTAIR and DNA methylation help eradicate OCSCs and block disease recurrence (180). In addition, several natural agents could target multiple signaling pathways. For instance, berberine was proved to target both EGFR and ErbB2. Berberine inhibited migration and invasion of ovarian cancer cells (181).

**Table 1:** Clinical trials of multi-immunotherapy in ovarian cancer.

Number	Clinical trial identifier	Targets	Responsible party	Status
**1**	NCT04024878	Nivolumab: PD-1 inhibitor NeoVax: 20 peptides and Poly-ICLC	Dana-Farber Cancer Institute	Recruiting
**2**	NCT05479045	Nivolumab: PD-1 inhibitor NY-ESO-1 Peptide vaccine	Georgetown University	Not yet recruiting
**3**	NCT02737787	Nivolumab: PD-1 inhibitor WT1 Vaccine NY-ESO-1 Vaccine	Memorial Sloan Kettering Cancer Center	Active, not recruiting
**4**	NCT05044871	Tislelizumab: PD-1 inhibitor Pamiparib: PARP inhibitor Bevacizumab: Anti-VEGF antibody	Tongji Hospital	Not yet recuiting
**5**	NCT03806049	Dostarlimab: PD-1 inhibitor Niraparib: PARP inhibitor Bevacizumab: Anti-VEGF antibody	Nordic Society of Gynaecological Oncology - Clinical Trials Unit	Withdrawn
**6**	NCT03602859	Dostarlimab: PD-1 inhibitor Niraparib: PARP inhibitor	Tesaro, Inc.	Active, not recruiting
**7**	NCT03955471	Dostarlimab: PD-1 inhibitor Niraparib: PARP inhibitor	Tesaro, Inc.	Terminated
**8**	NCT05467670	Pembrolizumab: PD-1 inhibitor ALX148: CD47 inhibitor	University of Pittsburgh	Not yet recuiting
**9**	NCT03596281	Pembrolizumab: PD-1 inhibitor Bevacizumab: Anti-VEGF antibody	Cancer Campus, Grand Paris	Active, not recuiting
**10**	NCT02537444	Pembrolizumab: PD-1 inhibitor Acalabrutinib: Bruton tyrosine kinase inhibitor	Acerta Pharma BV	Completed
**11**	NCT05188781	Pembrolizumab: PD-1 inhibitor Anlotinib: TKI	The Affiliated Hospital of Qingdao University	Completed
**12**	NCT03734692	Pembrolizumab: PD-1 inhibitor Rintatolimod: TLR-3 agonist	University of Pittsburgh	Recruiting
**13**	NCT03275506	Pembrolizumab: PD-1 inhibitor Bevacizumab: Anti-VEGF antibody	ARCAGY/GINECO GROUP	Active, not recruiting
**14**	NCT04361370	Pembrolizumab: PD-1 inhibitor Olaparib: PARP inhibitor Bevacizumab: Anti-VEGF antibody	Yonsei University	Enrolling by invitation
**15**	NCT05271318	Pembrolizumab: PD-1 inhibitor TILT-123: oncolytic adenovirus	TILT Biotherapeutics Ltd.	Recruiting
**16**	NCT04417192	Pembrolizumab: PD-1 inhibitor Olaparib: PARP inhibitor	National Cancer Center Hospital East	Recruiting
**17**	NCT05116189	Pembrolizumab: PD-1 inhibitor Bevacizumab: Anti-VEGF antibody	Merck Sharp & Dohme LLC	Recruiting
**18**	NCT04068974	Camrelizumab: PD-1 inhibitor Apatinib: VEGFR inhibitor	Peking Union Medical College Hospital	Recruiting
**19**	NCT05145218	TQB2450: PD-1 inhibitor Anlotinib: TKI	Chia Tai Tianqing Pharmaceutical Group Co., Ltd.	Recruiting
**20**	NCT03574779	TSR-042: PD-1 inhibitor Niraparib: PARP inhibitor Bevacizumab: Anti-VEGF antibody	Tesaro, Inc.	Recruiting
**21**	NCT03294694	PDR001: PD-1 inhibitor Ribociclib: CDK inhibitor Fulvestrant: ER downregulator	Dana-Farber Cancer Institute	Terminated
**22**	NCT02891824	Atezolizumab: PD-L1 inhibitor Bevacizumab: Anti-VEGF antibody	ARCAGY/GINECO GROUP	Active, not recruiting
**23**	NCT03695380	Atezolizumab: PD-L1 inhibitor Niraparib: PARP inhibitor Cobimetinib: MEK inhibitor	Hoffmann-La Roche	Recruiting
**25**	NCT03394885	Atezolizumab: PD-L1 inhibitor Bevacizumab: Anti-VEGF antibody	Duke University	Completed
**26**	NCT03353831	Atezolizumab: PD-L1 inhibitor Bevacizumab: Anti-VEGF antibody	AGO Research GmbH	Active, not recruiting
**27**	NCT03292172	Atezolizumab: PD-L1 inhibitor RO6870810: BET inhibitor	Hoffmann-La Roche	Terminated
**28**	NCT02915523	Avelumab: PD-L1 inhibitor Entinostat: HDAC inhibitor	Syndax Pharmaceuticals	Completed
**29**	NCT03642132	Avelumab: PD-L1 inhibitor Talazoparib: PARP inhibitor	Pfizer	Completed
**30**	NCT03558139	Avelumab: PD-L1 inhibitor Magrolimab: Anti-CD47 antibody	Gilead Sciences	Completed
**31**	NCT02943317	Avelumab: PD-L1 inhibitor Defactinib: PYK2 inhibitor	Verastem, Inc.	Terminated
**32**	NCT03704467	Avelumab: PD-L1 inhibitor M6620: ATR inhibitor	EMD Serono Research & Development Institute, Inc.	Completed
**33**	NCT03737643	Durvalumab: PD-L1 inhibitor Olaparib: PARP inhibitor Bevacizumab: Anti-VEGF antibody	AstraZeneca	Recruiting
**34**	NCT04742075	Durvalumab: PD-L1 inhibitor Olaparib: PARP inhibitor UV1: Peptide vaccine	Nordic Society of Gynaecological Oncology - Clinical Trials Unit	Recruiting
**35**	NCT02431559	Durvalumab: PD-L1 inhibitor Motolimod: TLR8 agonist	Ludwig Institute for Cancer Research	Completed
**36**	NCT02764333	Durvalumab: PD-L1 inhibitor TPIV200: A Multi-Epitope Anti-Folate Receptor Vaccine	Memorial Sloan Kettering Cancer Center	Completed
**37**	NCT03899610	Durvalumab: PD-L1 inhibitor Tremelimumab: CTLA-4 inhibitor	Yonsei University	Recruiting
**38**	NCT03699449	Durvalumab: PD-L1 inhibitor Olaparib: PARP inhibitor Cediranib: VEGFR inhibitor Tremelimumab: CTLA-4 inhibitor	Yonsei University	Recruiting
**39**	NCT03249142	Durvalumab: PD-L1 inhibitor Tremelimumab: CTLA-4 inhibitor	ARCAGY/GINECO GROUP	Active, not recruiting
**40**	NCT04015739	Durvalumab: PD-L1 inhibitor Bevacizumab: Anti-VEGF antibody Olaparib: PARP inhibitor	ARCAGY/GINECO GROUP	Active, not recruiting
**41**	NCT03430518	Durvalumab: PD-L1 inhibitor Eribulin: microtubule-targeting agent	Icahn School of Medicine at Mount Sinai	Completed
**42**	NCT04644289	durvalumab: PD-L1 inhibitor Olaparib: PARP inhibitor	AGO Research GmbH	Recruiting
**43**	NCT05422183	Envafolimab: PD-L1 inhibitor Lenvatinib: TKI	Zhongda Hospital	Not yet recruiting
**44**	NCT05130515	Niraparib: PARP inhibitor Anlotinib: TKI	Sun Yat-Sen Memorial Hospital of Sun Yat-Sen University	Not yet recruiting
**45**	NCT03783949	Niraparib: PARP inhibitor Ganetespib: Hsp90 inhibitor	Universitaire Ziekenhuizen Leuven	Active, not recruiting
**46**	NCT05198804	Niraparib: PARP inhibitor ZN-c3: Wee1 inhibitor	K-Group Beta	Recruiting
**47**	NCT05183984	Niraparib: PARP inhibitor Bevacizumab: Anti-VEGF antibody	ARCAGY/GINECO GROUP	Recruiting
**48**	NCT03895788	Niraparib: PARP inhibitor Brivanib: VEGFR and FGFR inhibitor	Hunan Cancer Hospital	Unkonwn
**49**	NCT04826198	Niraparib: PARP inhibitor AsiDNA: DNA Repair Inhibitor	Gustave Roussy, Cancer Campus, Grand Paris	Recruiting
**50**	NCT04149145	Niraparib: PARP inhibitor M4344: ATR inhibitor	University of Alabama at Birmingham	Not yet recruiting
**51**	NCT03944902	Niraparib: PARP inhibitor CB-839: Glutaminase inhibitor	University of Alabama at Birmingham	Terminated
**52**	NCT04734665	Niraparib: PARP inhibitor Bevacizumab: Anti-VEGF antibody	Yonsei University	Recruiting
**53**	NCT04376073	Niraparib: PARP inhibitor Anlotinib: TKI	Sun Yat-sen University	Recruiting
**54**	NCT04267939	Niraparib: PARP inhibitor Elimusertib: ATR inhibitor	Bayer	Recruiting
**55**	NCT03326193	Niraparib: PARP inhibitor Bevacizumab: Anti-VEGF antibody	Tesaro, Inc.	Active, not recruiting
**56**	NCT02354131	Niraparib: PARP inhibitor Bevacizumab: Anti-VEGF antibody	Nordic Society of Gynaecological Oncology - Clinical Trials Unit	Completed
**57**	NCT05009082	Niraparib: PARP inhibitor Bevacizumab: Anti-VEGF antibody	AGO Study Group	Not yet recruiting
**58**	NCT05170594	Fluzoparib: PARP inhibitor Bevacizumab: Anti-VEGF antibody	The Second Affiliated Hospital of Shandong First Medical University	Recruiting
**59**	NCT04517357	Fluzoparib: PARP inhibitor Apatinib: VEGFR inhibitor	Jiangsu HengRui Medicine Co., Ltd.	Recruiting
**60**	NCT05479487	Fluzoparib: PARP inhibitor Apatinib: VEGFR inhibitor	Fudan University	Not yet recruiting
**61**	NCT04229615	Fluzoparib: PARP inhibitor Apatinib: VEGFR inhibitor	Jiangsu HengRui Medicine Co., Ltd.	Active, not recruiting
**62**	NCT04669002	Olaparib: PARP inhibitor EP0057: NDC	Ellipses Pharma	Recruiting
**63**	NCT02889900	Olaparib: PARP inhibitor Cediranib: VEGFR inhibitor	AstraZeneca	Completed
**64**	NCT03117933	Olaparib: PARP inhibitor Cediranib: VEGFR inhibitor	University of Oxford	Active, not recruiting
**65**	NCT03278717	Olaparib: PARP inhibitor Cediranib: VEGFR inhibitor	NCT03278717	Recruiting
**66**	NCT02681237	Olaparib: PARP inhibitor Cediranib: VEGFR inhibitor	University Health Network, Toronto	Completed
**67**	NCT04729387	Olaparib: PARP inhibitor Alpelisib: PI3K inhibitor	Novartis Pharmaceuticals	Recruiting
**68**	NCT02340611	Olaparib: PARP inhibitor Cediranib: VEGFR inhibitor	University Health Network, Toronto	Completed
**69**	NCT02855697	Olaparib: PARP inhibitor Cediranib: VEGFR inhibitor	The Christie NHS Foundation Trust	Completed
**70**	NCT03314740	Olaparib: PARP inhibitor Cediranib: VEGFR inhibitor	Mario Negri Institute for Pharmacological Research	Unkonwn
**71**	NCT01623349	Olaparib: PARP inhibitor BKM120: PI3K inhibitor BYL719: PI3K inhibitor	Dana-Farber Cancer Institute	Completed
**72**	NCT02571725	Olaparib: PARP inhibitor Tremelimumab: CTLA-4 inhibitor	New Mexico Cancer Care Alliance	Active, not recruiting
**73**	NCT05494580	Pamiparib: PARP inhibitor Surufatinib: TKI	Sun Yat-sen University	Not yet recruiting
**74**	NCT00130520	Bevacizumab: Anti-VEGF antibody Erlotinib: EGFR inhibitor	University of Arizona	Completed
**75**	NCT04938583	Bevacizumab: Anti-VEGF antibody Oregovomab: Anti-CA125 antibody	Korean Cancer Study Group	Recruiting
**76**	NCT01551745	Bevacizumab: Anti-VEGF antibody Vigil™ Vaccine	Gradalis, Inc.	Completed
**77**	NCT01202890	Bevacizumab: Anti-VEGF antibody Lenalidomide: Immunomodulatory drug	New Mexico Cancer Care Alliance	Terminated
**78**	NCT01091259	Bevacizumab: Anti-VEGF antibody Irinotecan: Topoisomerase inhibitor	NYU Langone Health	Completed
**79**	NCT05113368	Regorafenib: Multi-kinase inhibitor Fulvestrant: ER degrader	Case Comprehensive Cancer Center	Not yet recruiting
**80**	NCT04625270	VS-6766: Dual RAF/MEK Inhibitor Defactinib: FAK Inhibitor	Verastem, Inc.	Recruiting
**81**	NCT01936363	Pimasertib: MEK inhibitor SAR245409: PI3K inhibitor	EMD Serono	Completed
**82**	NCT04998760	ATG-008: mTORC1/2 inhibitor ATG-010: Selective inhibitor of nuclear export compound	Chongqing University Cancer Hospital	Not yet recruiting
**83**	NCT05057715	VCN-01: Oncolytic adenovirus huCART-meso Cells	University of Pennsylvania	Recruiting
**84**	NCT02019524	E39: peptide vaccine J65: peptide vaccine	San Antonio Military Medical Center	Completed
**85**	NCT00003386	BCG vaccine autologous tumor cell vaccine	Sidney Kimmel Cancer Center at Thomas Jefferson University	Terminated
**86**	NCT02055690	Pazopanib: VEGFR inhibitor Fosbretabulin: Microtubule-targeting agent	The Christie NHS Foundation Trust	Terminated
**87**	NCT00408590	carcinoembryonic antigen-expressing measles virus oncolytic measles virus encoding thyroidal sodium iodide symporter	Mayo Clinic	Completed
**88**	NCT00799110	Dendritic Cell/Tumor Fusion Vaccine GM-CSF	Beth Israel Deaconess Medical Center	Active, not recruiting
**89**	NCT00181688	Iressa: EGFR inhibitor Arimidex: Aromatase inhibitor	Massachusetts General Hospital	Completed

PD-1, Programmed Cell Death Ligand 1; NY-ESO-1, New York esophageal squamous cell carcinoma-1; WT1, Wilms’ tumour 1; PARP, Poly (ADP-ribose) polymerase; VEGF, Vascular endothelial growth factor; TKI, tyrosine kinase inhibitor; TLR, Toll-like receptors; ER, Estrogen receptor; CDK, Cyclin-dependent kinase; PD-L1, Programmed cell death ligand 1; MEK, Mitogen-activated protein kinase; BET, Bromodomain and extraterminal domain; HDAC, Histone deacetylase; PYK2, Proline-rich tyrosine kinase 2; ATR, Ataxia-telangiectasia and Rad3-related protein; CTLA-4, Cytotoxic T-lymphocyte-associated protein 4; Hsp90, Heat shock protein 90; Wee1, Wee1-like protein kinase; FGFR, Fibroblast growth factor receptor; NDC, Nanoparticle-drug conjugate; PI3K, Phosphoinositide 3-kinase; EGFR, Epidermal Growth Factor Receptor; CA125, carbohydrate antigen 125; RAF, Rapidly accelerated fibrosarcoma; FAK, Focal adhesion kinase; mTOR, Mechanistic target of rapamycin.

To conclude, multi-immunotherapies of ovarian cancer are far from fully elucidated. Future studies should focus on fully recognizing immunogenic characteristics, developing biomarkers, and selecting eligible patients. Multi-immunotherapy is supposed to combine immunotherapies rationally while minimizing toxicities.

## Author contributions

XYH wrote the initial draft of manuscript. CB and XZ revised the manuscript. TY reviewed and approved content. All authors contributed to the article and approved the submitted version.

## Funding

This research was funded by the National Natural Science Foundation of China, grant number No. 81821002; Sichuan Science and Technology Program, grant number 2021YJ0011.

## Acknowledgments

Images were created with Biorender.com.

## Conflict of interest

The authors declare that the research was conducted in the absence of any commercial or financial relationships that could be construed as a potential conflict of interest.

## Publisher’s note

All claims expressed in this article are solely those of the authors and do not necessarily represent those of their affiliated organizations, or those of the publisher, the editors and the reviewers. Any product that may be evaluated in this article, or claim that may be made by its manufacturer, is not guaranteed or endorsed by the publisher.
